# Growth monitoring and promotion service utilization and its associated factors among mothers of children under two years in Ethiopia: a systematic review and meta-analysis

**DOI:** 10.1186/s12887-024-04946-1

**Published:** 2024-07-19

**Authors:** Yilkal Simachew, Arsema Abebe, Amanuel Yoseph, Berhan Tsegaye, Gedion Asnake, Hawa Hassen Ali, Rekiku Fikre

**Affiliations:** 1 School of Public Health, College of Medicine and Health Science, Hawassa University, Hawassa, Ethiopia; 2 Department of Public Health Nutrition and Dietetics, School of Public Health, Addis Ababa University, Addis Ababa, Ethiopia; 3 Department of Midwifery, College of Medicine and Health Sciences, Hawassa University, Hawassa, Ethiopia; 4 Pharma Health Science College, Hawassa, Ethiopia

**Keywords:** Growth monitoring and promotion, Under two years children, Ethiopia

## Abstract

**Background:**

Growth monitoring and promotion (GMP) is a nutritional intervention designed to identify and address growth faltering before a child’s nutritional status deteriorates into severe malnutrition. Despite GMP being recognized as a priority in Ethiopia’s national nutrition program, there is no national aggregated figure to show the extent of GMP service utilization. Therefore, this systematic review and meta-analysis aimed to assess GMP service utilization and associated factors in Ethiopia.

**Methods:**

A systematic literature search was conducted using PubMed/MEDLINE, CINAHL, Hinari, EMBASE, Scopus, and grey literature sources like Google Scholar, WorldCat, and Institutional repository. The Joanna Briggs Institution (JBI) quality assessment tool was used to appraise the quality of the articles, and articles scoring > 50% were included in the analysis. The pooled prevalence and odds ratio of associated factors with 95%CI was computed using STATA version 16. A random-effect model was employed to estimate the effect size, and I-squared statistics and Egger’s test were used to assess heterogeneity and identify potential publication bias, respectively. Subgroup analysis was conducted with publication year, sample size, and region to identify the source of heterogeneity.

**Results:**

Nine studies with 4,768 study participants were included in this meta-analysis. The overall pooled utilization of GMP service among children under two years of age in Ethiopia was 23.21% (95% CI: 16.02, 30.41, I^2^ = 97.27% & *P* = 0.0001). Mothers who received counselling on GMP service (OR = 3.16 (95%CI: 2.49-4.00), parents who use family health card (FHC) (OR = 3.29 (95%CI: 1.49–7.28), and mother who use postnatal care (OR = 3.93 (95%CI: 2.40–6.42), and Anti natal care (OR = 3.15 (95%CI: 1.29–7.69) were the factors associated with GMP service utilization among children under two years of age.

**Conclusions:**

The utilization of GMP services among children under the age of two in Ethiopia remains inadequate. Therefore, it is crucial to provide health education and counselling focusing on GMP to the mothers/caregivers of the child and encourage utilization of FHC. In addition, integrating GMP with other maternal health services should be promoted.

**Supplementary Information:**

The online version contains supplementary material available at 10.1186/s12887-024-04946-1.

## Introduction

Childhood malnutrition is a significant worldwide public health concern. In 2022, approximately 148.1 million children under the age of five were stunted, and 45 million children were wasted. Sub-Saharan Africa, in particular, carries a substantial burden of all types of undernutrition [1]. Ethiopia is among the nations significantly affected by various types of malnutrition.

Despite significant progress has been made in Ethiopia over recent decades, there is still much work to be done to reach the nutrition targets set for 2025 by the World Health Assembly (WHA) and the Sustainable Development Goals (SDG) for 2030 [2, 3]. Child undernutrition continues to be a serious public health issue throughout the country [4]. As per the 2019 Ethiopia Demographic and Health Survey, over one-third (37%) of children under the age of five suffer from stunted growth, 21% are underweight, and 7% are wasted [5].

Insufficient growth and development during early childhood increase the risk of severe infections and vulnerability to common childhood illnesses, contributing to almost half of all deaths in children under the age of five [6]. In addition, malnourished children are more likely to face long-term consequences like reduced productivity, quality of life, and inter-generational impact [7]. Therefore, optimizing child growth and nutrition is a key strategy for reducing undernutrition in this age group for addressing the under-five mortality rate as part of the Sustainable Development Goal [8]. In response to this, WHO recommends the inclusion of Growth monitoring and promotion (GMP) as a vital component of child health and nutrition strategies [9].

Growth monitoring and promotion (GMP) is a nutritional intervention to prevent and manage malnutrition. It involves monitoring a child’s growth through regular weight and height measurements, comparing the child’s growth to a standard growth chart, interpreting the growth patterns, and taking appropriate actions such as providing nutritional counselling, supplements, or conducting health examinations if necessary [10]. GMP serves as an early warning system, identifying and addressing growth faltering before a child’s nutritional status deteriorates into severe malnutrition. Many agree the GMP is one of the cost-effective strategies to reduce child malnutrition when both growth charting and promotion activities are appropriately integrated [11].

Ethiopia has endorsed global and national commitments to see children free from undernutrition. In 2015, the Ethiopian government launched the “Seqota Declaration” to eliminate stunting in children under the age of two by the year 2030. The National Nutrition Programme of Ethiopia also prioritizes addressing nutrition issues during the first two years of a child’s life [12, 13]. It seeks to accomplish this goal by implementing a range of evidence-based, nutrition-specific interventions, including Growth Monitoring and Promotion (GMP) [14].

Despite the recognized importance of GMP in preventing undernutrition and increased attention from the Ethiopian government, data shows that the utilization of GMP services remains limited in the country.

Despite the recognized importance of GMP in preventing undernutrition and increased attention from the Ethiopian government, data shows that GMP service utilization remains limited in the country [14]. This is supported by studies conducted in different regions of Ethiopia, where GMP service utilization ranges from 13.4% in Debre-Berhan to 38.9% in northwest Ethiopia [15–22]. Several factors contributed to the low GMP service utilization in Ethiopia. Accessibility issues, particularly in rural and remote areas, hinder service reach. A lack of awareness and education about GMP’s benefits, coupled with inconsistent and poorly resourced services, further discourages utilization [14–20]. Previous studies shown that socioeconomic status, the educational level of caregivers, and access to healthcare facilities significantly influence GMP service utilization. Additionally, the availability of information about the importance of GMP, maternal knowledge, the intention of mothers to use the service, and the quality of healthcare services can impact GMP utilization [15–22].

Despite GMP being recognized as a priority in Ethiopia’s national nutrition program, there is no single national aggregated figure available to show the extent of GMP service utilization. Therefore, this systematic review and meta-analysis aimed to estimate the overall pooled utilization of GMP service and its associated factors among under two-year-old children in Ethiopia. Hence, the result of this study will be valuable for nutrition program implementers, policymakers, stakeholders, and health professionals.

## Method

The protocol for this systematic review and meta-analysis was registered on the PROSPERO international database with a registration ID of CRD42023409013. For reporting, we followed the Preferred Reporting Items for Systematic Reviews and Meta-Analyses (PRISMA-2020) guideline [23].

### Information sources and search strategy

A comprehensive search was conducted to gather all relevant published studies and grey literature. The search was conducted electronically using databases such as PubMed/MEDLINE, EMBASE, Scopus, CINAHL, Google Scholar, Hinari, Worldcat, and repositories from Ethiopian universities. Furthermore, a search was conducted through the reference list of all identified articles to uncover additional relevant studies. The study articles were searched from April 01 to June 30, 2023. The systematic searching strategy was conducted by two authors independently using the combination of the following keywords and Boolean operators: (“Growth monitoring and promotion [Mesh]” OR “Growth monitoring*” OR GMP) AND (“Under two children” OR Child [Mesh] OR Children* OR “under two children” OR “less than 2 years children” or Infant OR “0–23 months children”) AND (Ethiopia OR Ethiopian OR “Regions of Ethiopia”).

### Inclusion and exclusion criteria

All observational studies (cross-sectional, case-control, cohort studies), with prevalence and/or associated factors of GMP service utilization in Ethiopia were included. Only articles conducted in English language were considered regardless of its publication from 1980 to the time of searching on June 30, 2023. Whereas, studies without full text and purely qualitative studies were excluded.

### Outcome measurement

This meta-analysis measures two main outcomes. The primary outcome of the study was utilization of GMP service among children under the age of two years. It was computed by dividing the number of GMP utilization by the total number of under 2-year children included in the study. Literatures operationalize utilization of GMP service when a child has a history of GMP service utilization with respect to their age, at least once for 0 months, twice for 1–3 months, five times for 4–11 months, and four times per year for 12–23 months [16–22].

The second outcome of this meta-analysis was the associated factors of GMP service utilization. To measure the relation of variables and GMP service utilization, odd ratio was used. The odd ratio was computed, after extracting the relevant data from the included studies using a two-by-two table.

### Study selection and quality appraisal

All articles retrieved from different database were exported to the endnote reference manager to remove any duplicates. Two authors screened the title and abstract of the remaining articles independently and classified them as included, excluded and undecided. We resolved any discrepancy between the two authors through a discussion with the third author.

Two authors assessed the quality of eligible studies independently using the Joanna Briggs Institution (JBI) critical appraisal checklist adapted for cross-sectional and case-control studies, and those articles scoring above 50% were included in the analysis [24].

### Data extraction

From the eligible studies, necessary data was extracted using Microsoft excel. The following data were extracted using a data extraction sheet: author name, study area, region, publication year, study design, sample size, response rate, number of children utilized GMP service, and proportion of children who utilized GMP service. For the second outcome (associated factors of GMP service utilization), data were retrieved in the form of two-by-two tables (Additional file 1).

### Data analysis

All extracted data were imported to STATA version 16 for analysis. A random-effects model was applied due to the heterogeneity. The I^2^ statistics was used to assess the heterogeneity between the included studies. To identify the source of heterogeneity among studies that show heterogeneity, subgroup analysis and univariate meta-regression were conducted. The Funnel plot and Egger’s test were used to assess the publication bias. The pooled prevalence of GMP service utilization was estimated by random effect. The log odds ratio was used to determine the association of GMP service utilization and risk factors by the DerSimonian-Laird Random effects model (REM) for meta-analysis. Table and forest plots were used to summarize the findings of selected studies.

## Results

### Study searches and selection

**Fig. 1:**
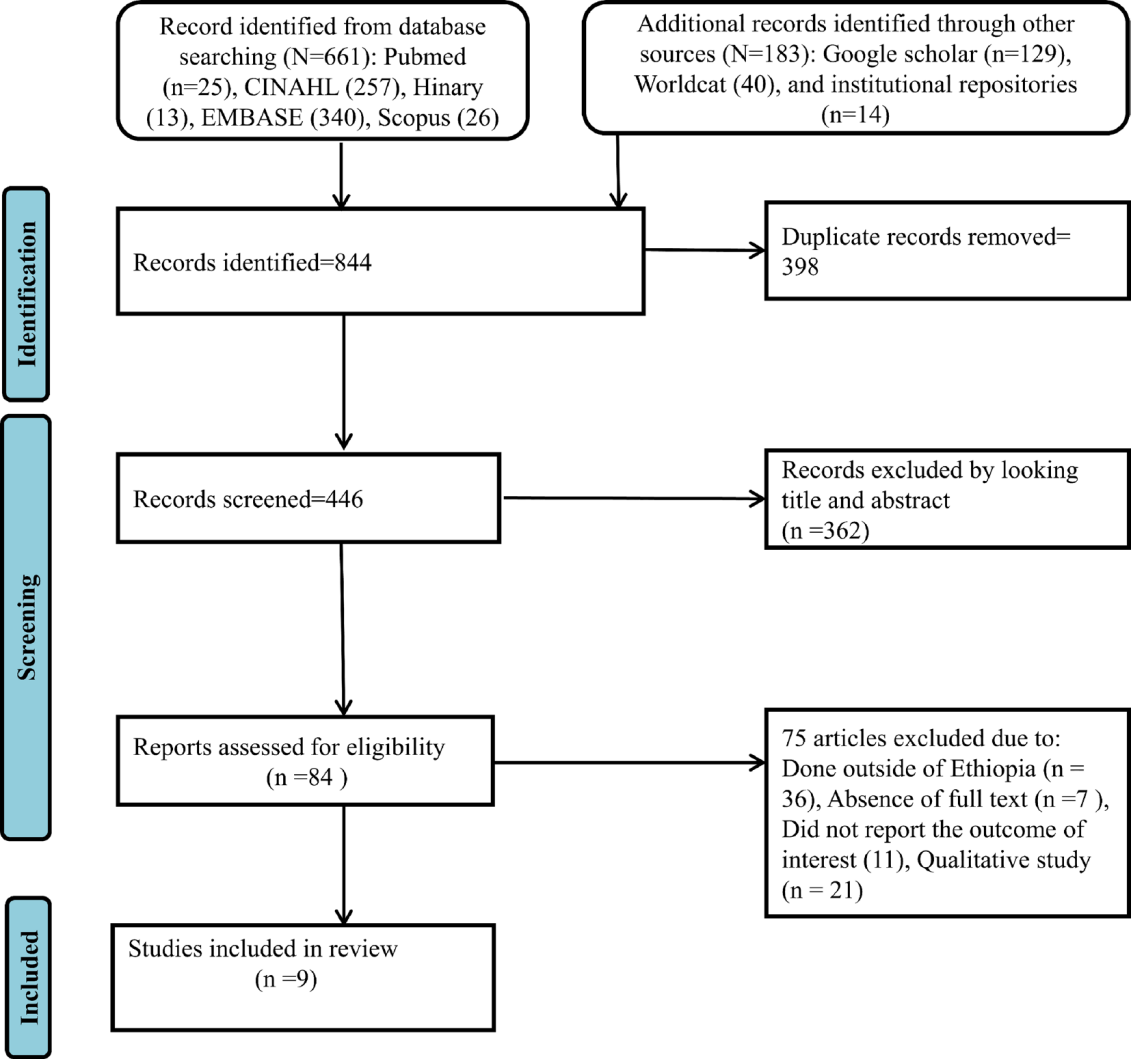
The PRISMA flow chart for the selection of studies for systematic review and meta-analysis

A total of 844 articles were identified through manual and electronic searches. Among these, 398 were removed by endnote reference software because of duplication. Then, 362 were excluded after the screening of the title and abstract. A total of 84 full articles were assessed based on the eligibility criteria, and 75 were excluded for the following reasons: 36 of the studies were conducted outside of Ethiopia, 7 of the articles were without full text, 11 articles did not report the outcome of interest, and 21 of the study were qualitative study. The final analysis was conducted among nine studies that met the eligibility criteria [15–22, 25] (Fig. 1). Among eight studies, eight of them reported both the prevalence and associated factors of GMP service utilization, while one study reported only the risk factors (OR).

### Characteristics of included studies

A total of 9 studies with 4,768 study participants were included in this meta-analysis. Among nine eligible studies, eight studies were community-based cross-sectional studies, and one was a case-control study. Four of the studies were conducted in the Southern Nations, Nationalities, and Peoples’ Region (SNNPR), three studies were in the Amhara region, and the rest were in the Oromia and Afar regions. The smallest sample size was 354 from a study conducted in SNNPR, and the largest was 965 from a study conducted in the Oromia region. The lowest prevalence of GMP service utilization (10.85%) was reported in the SNNPR, and the highest (38.86%) was in the Amhara region (Table 1).

**Table 1:** Characteristics of eligible studies

Author and publication year	Region	Study design	Sample size	Sampling procedure	Response rate (%)	Case	Prevalence	Quality score
Kiros S et al. 2023 [19]	Afar	Cross-sectional	396	Simple random	95	63	15.91	75
Sahle LD. 2017 [20]	SNNPR	Cross-sectional	507	Simple random	93	55	10.85	62.5
Endale G et al. 2020 [16]	SNNPR	Cross-sectional	443	Simple random	96	143	32.28	75
Tufa G et al. 2022 [21]	SNNPR	Cross-sectional	354	Simple random	95.2	89	25.14	87.5
Yeshaneh A et al. 2020 [22]	Amhara	Cross-sectional	561	Simple random	98	218	38.86	87.5
Girma D et al. 2018 [18]	Oromia	Cross-sectional	965	Systematic	92.6	317	32.85	75
Feleke FW et al. 2017 [17]	SNNPR	Cross-sectional	782	Multistage	95	132	16.88	87.5
Ahmed F et al. 2020 [15]	Amhara	Cross-sectional	402	Multistage	95.4	54	13.43	75
Dagne S et al. 2020 [25]	Amhara	Case-control	358	Multistage	98.6	-	-	70

### Pooled GMP service utilization among under two years children in Ethiopia

**Fig. 2:**
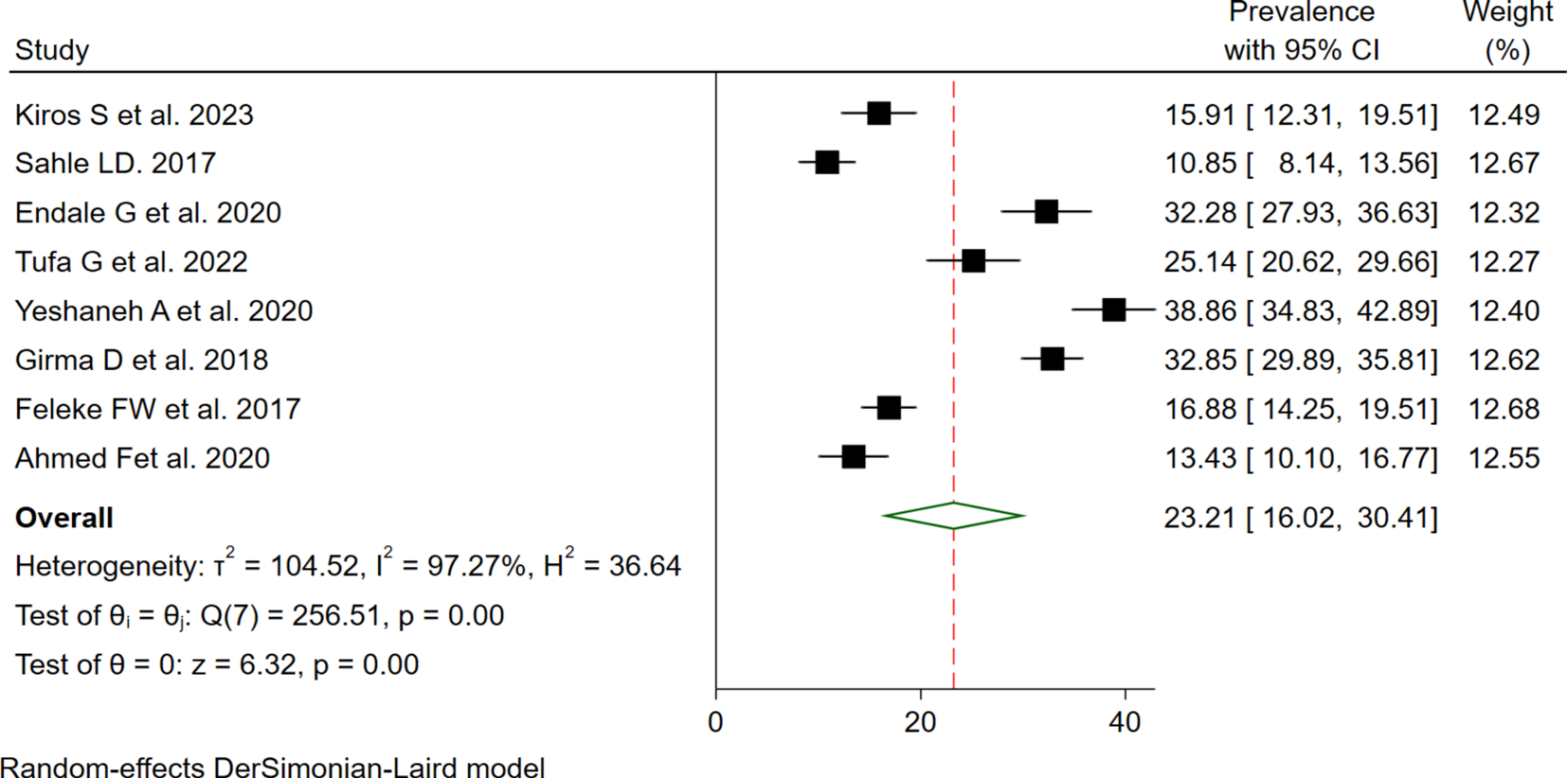
Forest plot diagram indicates the pooled utilization of GMP service among under two-year children in Ethiopia, 2023

**Fig. 3:**
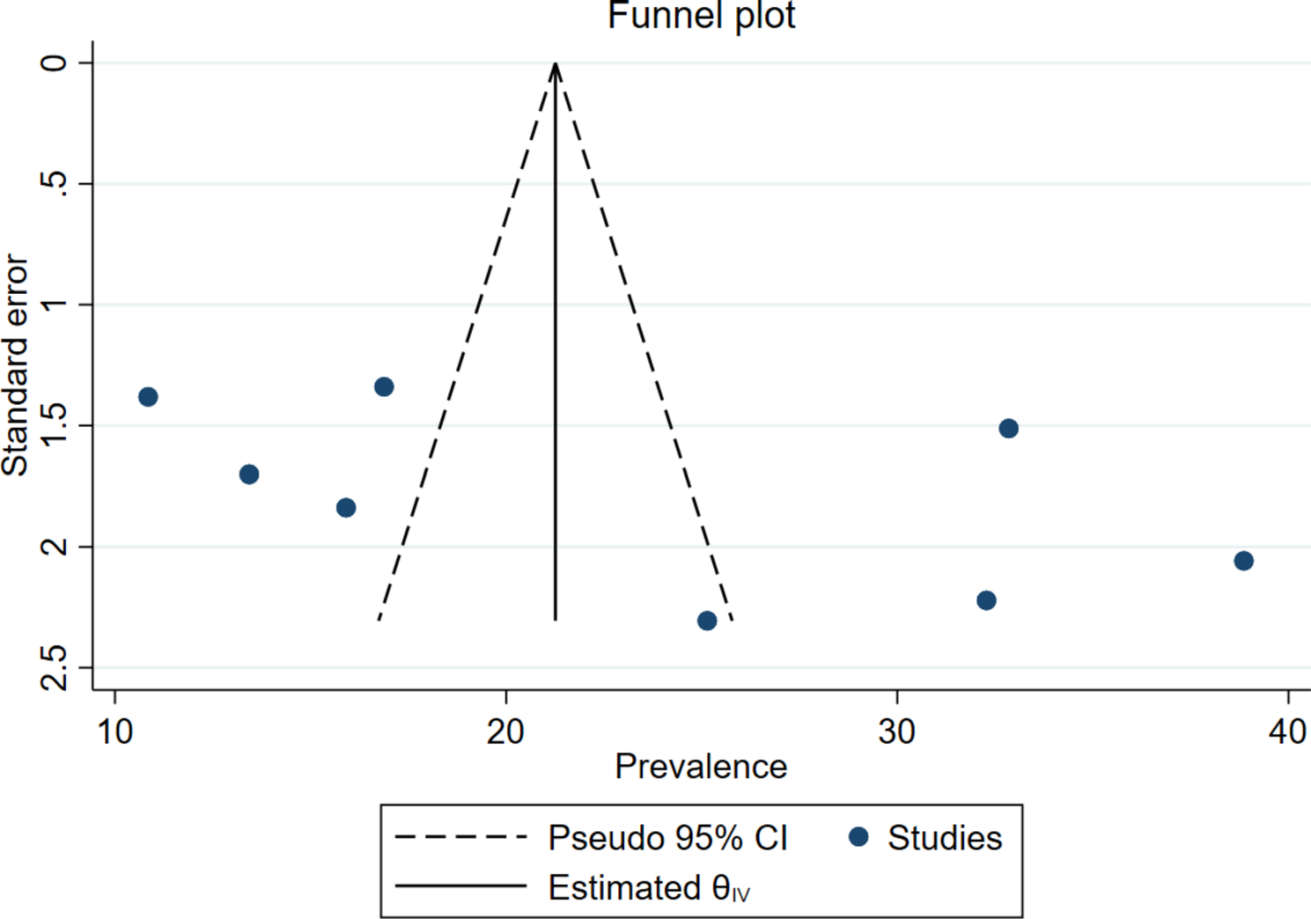
Funnel plot of the pooled utilization of GMP service among under two children in Ethiopia, 2023

Of nine eligible studies, eight studies were used to determine the pooled utilization of GMP service, because one of the studies was case-control that provided only risk factors (OR). The overall pooled utilization of GMP service among under two years children in Ethiopia was 23.21% (95% CI: 16.02, 30.41, I^2^ = 97.27% & *P* = 0.0001) (Fig. 2). The I^2^ statistic shows significant heterogeneity among studies. To check the source of variation univariate meta-regression was conducted using publication year, sample size, and response rate. However, none of them show statistically significant source of heterogeneity among the included articles (Table 2). Publication bias was checked using a funnel plot and Eggers test. The funnel plot shows the absence of publication bias with symmetrical distribution (Fig. 3). Additionally, the Egger’s test p-value was 0.117, which shows the absence of publication bias for estimating the prevalence of GMP service utilization among under two-year children.

**Table 2:** Univariate meta-regression to identify factors associated with heterogeneity of GMP service utilization among under two years children in Ethiopia, 2023

Variables	Coefficient	Standard error	*P*-value
Sample size	0 0.0346298	0.0286225	0.226
Study year	1.69287	2.709052	0.532
Response rate	3.76124	2.833091	0.184

### Subgroup analysis

To identify the source of heterogeneity among the included studies, we have conducted subgroup analysis by region, publication year, and sample size. The subgroup analysis shows that there is no significant variation between group of regions, sample size and study year (Table 3).

**Table 3:** Subgroup pooled prevalence of GMP service utilization among under two-year children by region, publication year, and sample size, 2023

Variables	Characteristics	Included studies	Estimate with (95%CI)	I^2^
Sample size	<=500	4	21.60 (13.21, 29.99)	94.58%
	> 500	4	24.80 (12.60, 37.01)	98.49%
Study year	<=2018	3	20.18 (7.78, 32.58)	98.35%
	> 2018	5	25.08 (15.32, 34.84)	96.77%
Region	SNNPR	4	21.14 (12.53, 29.75)	96.17%
	Ahmara	2	26.12 (1.20, 51.04)	98.90%
	Oromia	-	-	-
	Afar	-	-	-

### Factor associated with GMP service utilization among under two children

From nine included studies, the following seven factors were eligible to conduct the meta-analysis: place of delivery, knowledge of GMP, ANC service utilization, use of family health card (FHC), educational status of child’s mother, GMP counselling, and utilization of PNC. From this, four show a statistically significant association with GMP service utilization; mother who received counselling on GMP services, parents who use FHC, and mothers who use PNC and ANC.

**Fig. 4:**
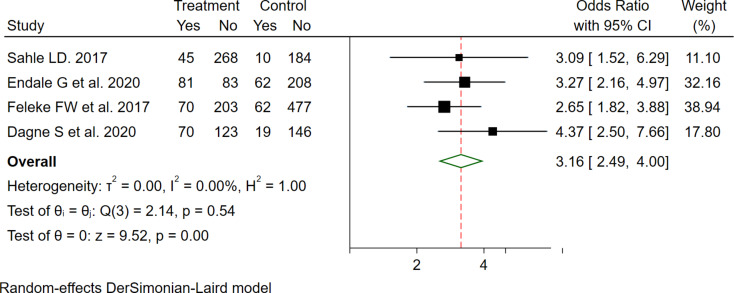
The pooled odd ratio of the association between counseling on GMP and GMP service utilization among under two children in Ethiopia, 2023

This meta-analysis shows that mothers/caregivers who received GMP counselling were 3.16 times more likely to utilize GMP services than those who did not receive counselling (OR = 3.16 (95%CI: 2.49-4.00), with no evidence of heterogeneity (I2 = 0.0) (Fig. 4).

**Fig. 5:**
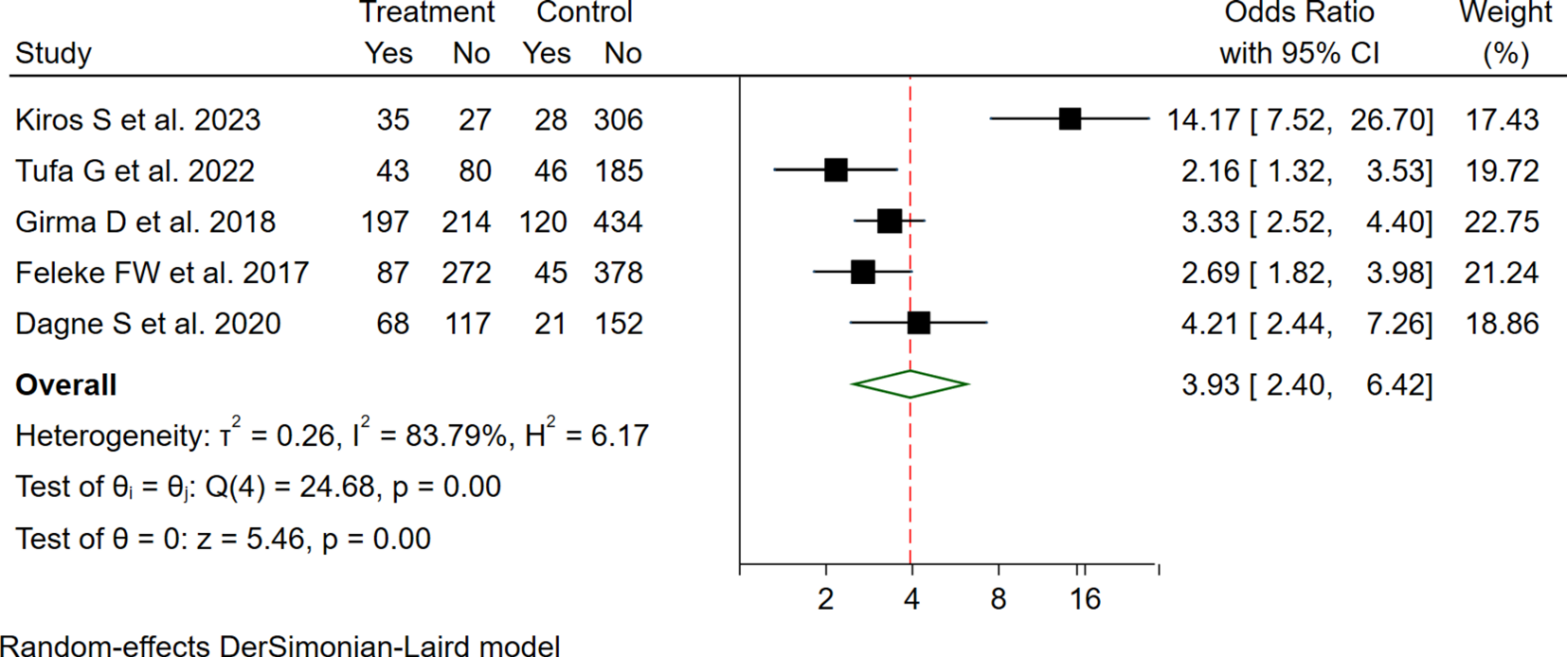
The pooled odd ratio of the association between maternal PNC use and GMP service utilization among under two children in Ethiopia, 2023

Based on a pooled analysis of four studies, mothers who received PNC service for the indexed child were more likely to utilize GMP services than their counterparts (OR = 3.93 (95%CI: 2.40–6.42), with significant high heterogeneity (I2 = 83.79) (Fig. 5).

**Fig. 6:**
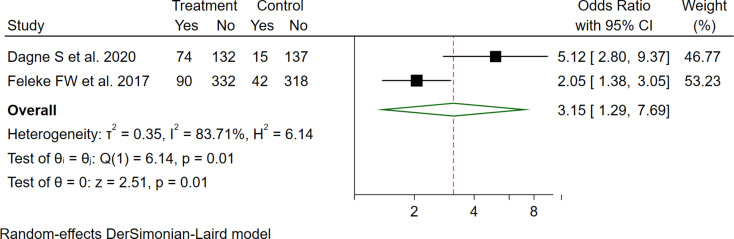
The pooled odd ratio of the association between maternal ANC use and GMP service utilization among under two children in Ethiopia, 2023

Similarly, mothers who had follow-up for ANC service were 3.15 times more likely to utilize GMP services for their child as compared to mothers who had no follow-up history for ANC service (OR = 3.15 (95%CI: 1.29–7.69) (Fig. 6).

**Fig. 7:**
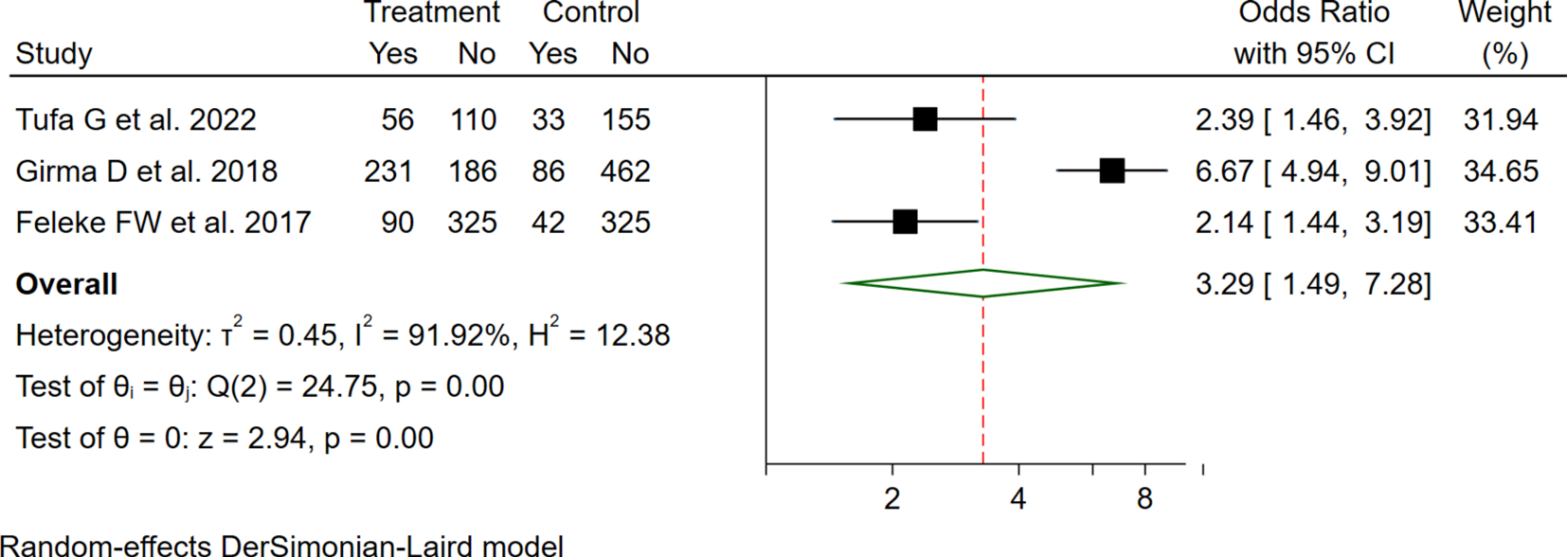
The pooled odd ratio of the association between FHC use and GMP service utilization among under two children in Ethiopia, 2023

The pooled effect of three studies also revealed that the odds of GMP service utilization was 3.29 times higher among mothers who used FHC as compared with those who did not (OR = 3.29 (95%CI: 1.49–7.28) (Fig. 7).

## Discussion

This review was conducted to estimate the overall pooled utilization of GMP service and its associated factors among under two-year-old children in Ethiopia. The pooled GMP service utilization among under two children was 23.21% (95% CI: 16.02, 30.41). Despite optimizing child growth and nutrition through GMP service utilization is a priority strategy to achieve the 2030 target of ending child undernutrition in Ethiopia, there is still a low level of GMP service utilization in Ethiopia. This suggests the need for strengthening efforts to promote GMP service utilization. This is consistent with a study done in Ghana (28.5%) [26]. However, these result is lower than studies conducted in Rwanda (33%) [27], and Lawra district in Ghana (70%) [28]. The difference could be explained by the variation in the population characteristics, study setting, accessibility to GMP services, the quality of health services and GMP program.

There is a positive association between mother who have received PNC and their utilization of GMP services for their children under the age of two. These findings agree with the study conducted in Malawi, and Benin, which reported that children whose mothers had PNC were more likely to get child health services [29, 30]. This is because PNC is one of the components of maternal and child health service, often including nutritional counselling and health education sessions. As a result, mothers with a history of PNC visits are more likely to be aware of the importance of GMP services for their children’s growth and development. Additionally, PNC visits offer an opportunity for health workers to promote and encourage the utilization of GMP services.

In addition, mothers who received ANC were more likely to utilize GMP services for their children than their counterparts. This finding is similar to the study done in Kenya [31]. This is because ANC visits typically include discussions on various aspects of maternal and child health, including nutrition and growth monitoring which empowers mothers with the knowledge and information they need to make informed decisions about their child’s health. This association underscores the necessity of promoting ANC service utilization among pregnant women. Additionally, health workers should be well-equipped to provide adequate information to pregnant women about the importance of GMP after delivery.

This meta-analysis also showed that mothers who used family health cards were more likely to utilize GMP services compared to those who did not. This finding is supported by a study conducted in Kenya [31]. Family health cards typically contain essential health information, including immunization schedules and milestones in a child’s growth. By using these cards, mothers have easy access to crucial information about their child’s health, which can motivate them to seek GMP services as part of their commitment to their child’s well-being. A family health card can serve as a reminder for mothers to seek healthcare services for GMP. In addition, service providers can use the cards as a tool to encourage and educate mothers about the significance of monitoring their child’s growth, which motivates mothers to GMP service utilization. This finding suggests that promoting the use of FHC can be an effective strategy to increase the utilization of GMP services and ultimately support child health and development.

In addition, mothers/caregivers who received counselling on GMP have a better chance of utilizing GMP services. This is consistent with a study conducted in Kenya [31]. This finding indicates that counselling plays a significant role in encouraging mothers to seek and utilize GMP service, because it equips a mother with the knowledge to make an informed decision. When mothers are aware of the positive impact of GMP on their children’s health and well-being, they are more likely to seek and engage in GMP services. Furthermore, counselling helps to address any misconceptions or concerns that mothers may have about GMP.

### Strength and limitation of the study

This study was the first systematic review and meta-analysis which showed the pooled utilization of GMP service and associated factors among under two years children in Ethiopia. However, the lack of studies from all regions of Ethiopia might limit the national representativeness of the study.

## Conclusion

Utilization of GMP service among under two years children in Ethiopia is still insufficient seven years prior to “Seqota Declaration”, a national initiative to eliminate stunting by the year 2030. Utilization of ANC service, PNC service, FHC, and counselling toward GMP were determinants of GMP service utilization. Therefore, it is crucial to provide health education and counselling that focus on GMP to the mothers/caregivers of the child. Besides that, it is advisable to encourage the utilization of FHC by the mother/caregiver of the children. Furthermore, the integration of GMP with other maternal health services (like ANC and PNC) should be promoted.

### Electronic supplementary material

Below is the link to the electronic supplementary material.

Supplementary Material 1

## Data Availability

The data that support the review findings of this study are included in the manuscript and with Additional files.

## Abbreviations

ANC: Ante Natal Care
EDHS: Ethiopian Demographic Health Survey
FHC: Family Health Card
GM: Growth Monitoring
GMP: Growth Monitoring and Promotion
PNC: Post Natal Care
SDG: Sustainable Developmental Goal
SNNPR: Southern Nation and Nationality People Regional State
